# The 12-Step Pathway to Spiritual Growth and Gratitude and Its Relationship With Well-Being Among the Members of Sexaholics Anonymous in Poland

**DOI:** 10.1007/s10943-023-01892-7

**Published:** 2023-08-31

**Authors:** Marcin Wnuk, Amy R. Krentzman, Edyta Charzyńska

**Affiliations:** 1 Department of Work and Organizational Psychology, Faculty of Psychology and Cognitive Sciences, Adam Mickiewicz University in Poznań, Szamarzewskiego 89/AB, 60-568 Poznań, Poland; 2 School of Social Work, University of Minnesota, 1404 Gortner Avenue, St Paul, MN 55108 USA; 3 Faculty of Social Sciences, Institute of Psychology, Institute of Pedagogy, University of Silesia in Katowice, Bankowa 12, 40-007 Katowice, Poland

**Keywords:** Spirituality, Gratitude, 12-step program, Compulsive sexual behavior disorder, Poland

## Abstract

Despite the well-established role of mutual-aid groups in addiction treatment, no research has yet explored the role of the 12-step pathway in spiritual growth and gratitude, or its direct and indirect relationships with well-being among individuals with compulsive sexual behavior disorders (CSBD). The purpose of the current study was to examine the number of 12 steps completed as an antecedent of spiritual growth and gratitude and its relationship with well-being among members of Sexaholics Anonymous (SA). The sample consisted of 80 individuals (72 men and 8 women) attending SA meetings in Poland. The study variables were measured using the Daily Spiritual Experiences Scale, Gratitude Questionnaire, Satisfaction with Life Scale, Positive and Negative Affect Schedule, and a single question regarding the number of 12 steps completed. A path analysis showed that the number of 12 steps completed was negatively and directly related to negative affect. Moreover, the number of 12 steps completed was related to higher spiritual growth, which was directly related to higher levels of life satisfaction and lower levels of negative affect, and indirectly, through gratitude, to higher levels of life satisfaction and positive affect. The results suggest that spiritual growth, rooted in the 12-step program, and its ability to increase gratitude, may promote recovery from CSBD.

## Introduction

### The 12-Step Program as an Antecedent of Spiritual Growth in Addictions

Although spirituality is a complex and broad construct, which makes it difficult to define, researchers have been trying to conceptualize it following the standards of scientific research and have reached some degree of consensus on the matter. Spirituality, as an inherent component of being human, is connected with the belief that there are aspects of human life that transcend physical reality and that there is more to life than what we see or fully understand (Underwood & Teresi, 2002). A spiritual person seeks answers to fundamental questions about life and looks for meaning and purpose in their lives in connectedness with others, oneself, nature, and the sacred or transcendent (Cook, 2004).

Spirituality might be considered within the context of a specific religion, it might be expressed as a profound and personal relationship with God / higher being, or it might have a more secular meaning for the ways in which it is associated with nature, arts, music, family, and community (Puchalski & Guenther, 2012). Therefore, although for some people, spirituality overlaps with religion, for others, it has nothing in common with it. Conversely, religion may be deprived of spirituality when religious practices are done automatically or instrumentally without meaning and engagement (Worthington & Aten, 2009).

In this study, we explored an aspect of spirituality—daily spiritual experiences—understood as an individual’s experiences, awareness, and relationship with the divine or transcendent in daily life (Underwood & Tedeschi, 2002). These are more secular expressions of spirituality not focused on beliefs or behaviors of a specific religious doctrine. Greater numbers of daily spiritual experiences may be understood to represent spiritual growth. Much previous research has shown that spirituality conceptualized in this way is associated with greater life satisfaction (Skarupski et al., 2013; Rudaz et al., 2019) and positive affect (Ellison & Fan, 2008; Van Cappellen et al., 2023), and less negative affect (Brelsford et al., 2020; Park & Slattery, 2012).

The topic of spirituality and its effects on the recovery of individuals with addictions have been extensively explored, especially for those with alcohol dependence and their partners participating in mutual-aid groups (Dermatis & Galanter, 2016; Krentzman et al., 2013; Wnuk, 2022b; Wnuk, 2023). Alcoholics Anonymous (AA) is a non-professional, egalitarian, effective form of treatment for alcohol dependence, serving as a prototype for other mutual-aid groups focused on recovering from drugs, gambling, sex, and other types of addictions (Kelly et al., 2020). AA philosophy considers alcoholism to be a progressive, chronic, incurable, and deadly disease rooted in spiritual decline and egocentrism, which can be overcome only through spiritual restoration and transformation as the result of following the 12-step program (AA, 1953, 2001). It also emphasizes the need to distinguish abstinence from sobriety, suggesting that abstinence by itself is not a sufficient factor for sobriety; sobriety is a broader concept understood as abstinence as well as leading a happy and satisfying life (AA, 2001). In previous studies, participation in mutual-aid groups for addictions correlated not only with longer abstinence (Krentzman et al., 2013; Zemore, 2007) and reduced symptoms of addiction (Galanter et al., 2013; Kaskutas et al., 2002) but also with higher levels of subjective well-being (Gomes & Hart, 2009; Schiff & Bargal, 2000; Wnuk, 2022a) and meaning in life (Zemansky, 2006).

As further support for the beneficial role of self-help groups in recovery from addictions, many studies have shown the positive effect of involvement in such groups on spiritual growth (Galanter et al., 2013; Krentzman et al., 2013, 2017; Montes & Tonigan, 2017; Wnuk, 2022a). For example, in Zemore’s (2007) longitudinal study, increases in 12-step involvement from baseline to follow-up predicted a higher probability of abstinence at follow-up, and increases in spirituality mediated this relationship. In another longitudinal study, spiritual growth, measured as gains in spiritual and religious practices, mediated the relationship between the frequency of AA attendance and the percentage of days abstinent (Montes & Tonigan, 2017). Involvement in AA has been shown to be associated with increases in daily spiritual experiences, private religious practices, forgiveness of others (Krentzman et al., 2013, 2017), and with positive religious coping (Krentzman et al., 2017). The current study aims to investigate the role of the 12-step pathway in the spiritual growth, gratitude, and subjective well-being of individuals with compulsive sexual behavior disorder (CSBD) who attend Sexaholics Anonymous (SA).

### Characteristics of CSBD

Currently, there is no research regarding the role of spirituality and religiousness in the spiritual growth of patients with CSBD (also known as “sexual addiction,” “sexual dependence,” “hypersexuality,” “problematic sexual behavior,” or “sexual impulsivity”). Individuals with CSBD exhibit distress or significant impairment in various important areas of functioning for six months or more due to a persistent pattern of failure to control intense and recurrent sexual impulses, urges, and/or thoughts, resulting in repetitive sexual behavior (Kraus et al., 2018). Behaviors such as masturbation, pornography use, cybersex, having multiple sexual partners and engaging in sexual fantasies are predominantly reported by patients with CSBD. Unsatisfying or unprotected sexual intercourse and having multiple sexual partners are most often indicated as problematic for this group (Wéry et al., 2016).

Despite being categorized as an impulse control behavior in the 11th revision of the International Classification of Diseases (ICD-11), the psychiatric classification of CSBD is still debated since the CSBD shares many similarities with addictive behaviors, such as clinical symptoms, etiology, neurocognitive mechanisms, sociodemographic correlates, and therapeutic interventions (Efrati et al., 2021). It is estimated that CSBD affects about 3–6% of adult population (Bőthe et al., 2019; Klein et al., 2014), with men comprising around 80% of patients seeking treatment (Kaplan & Krueger, 2010).

The forms of treatment for CSBD include emotion-focused therapy, cognitive-behavioral therapy, mindfulness-based therapy, or pharmacotherapy (Efrati & Gola, 2018; Holas et al., 2021). In Poland, mutual-aid groups are the leading form of treatment for patients with CSBD, and two 12-step recovery fellowships are active in the country (Wnuk & Charzyńska, 2022): SA and Sex and Love Addicts Anonymous (SLAA). These two groups are similar in their assumptions and the 12-step model of treatment. The main difference between them lies in the definition of sexual abstinence, with SA being more restrictive than SLAA, counting only heterosexual sex with one’s own spouse as acceptable sexual behavior constituting abstinence from sexual addiction.

Over the past decade, there has been a growing interest in the relationship between religiousness, spirituality, and CSBD. Research in this area has focused mostly on examining the role of religiousness in the course of CSBD, on exploring the function of moral incongruence in the relationship between religiousness and CSBD symptoms (Grubbs et al., 2015, 2017; Jennings et al., 2021), and on testing religiousness as a protective factor against internet pornography use (Baltazar et al., 2010; Wright, 2013). Studies on the relationship between spirituality and CSB are scarce, but those available have demonstrated the negative relationship between indicators of spiritual well-being and CSB and the positive relationship between CSB and aspects of spiritual struggles (see Jennings et al., 2021).

### Gratitude and Addiction

#### Spiritual Roots of Gratitude

Gratitude is a positive, pleasant emotion involving feelings of thankfulness related to appreciation for, or recognition of something positive (Emmons & Shelton, 2002). Gratitude has three dimensions: *state gratitude*, which is a momentary feeling of thankfulness or appreciation; *trait gratitude*, which is an overall disposition toward noticing and appreciating life’s positive aspects (Wood et al., 2010); and *gratitude practices*, which are intentional behaviors designed to cultivate gratitude, such as writing a gratitude list (Emmons & Stern, 2013).

In many religious systems and spiritual paths, gratitude is treated as a desired value and virtue (Carlisle & Tsang, 2013; Emmons & Kneezel, 2005). In a religious context, human beings should be grateful to God as a source of all good (Watkins et al., 2022). A grateful attitude to God, who is perceived as a benefactor, is enhanced by a cultural reciprocity norm (Gouldner, 1960). When a religious person feels the abundance of gifts received from God, their gratitude to God may magnify and amplify a grateful attitude toward other people by evoking the willingness to give back (Nelson et al., 2023). Moreover, spiritual experiences, which are often connected with a feeling of awe and beauty in life (Büssing et al., 2014), lead to the appreciation of life itself, other creatures, and material and immaterial gifts that a person receives from other people, nature, and the Universe (Olson et al., 2019).

Consistent with the above theoretical premises, many studies have shown that religious and spiritual involvement is a source of gratitude (Krause & Hayward, 2015; Lambert et al., 2009; Olson et al., 2019; Rosmarin et al., 2016; Tsang et al., 2012). For example, in a longitudinal study by Hardy et al. (2014), daily religious practices were positively associated with gratitude, forgiveness, and empathy through daily spiritual experiences. In four studies applying various methodologies, including longitudinal and experimental designs, Lambert et al. (2009) noted the effectiveness of prayer for increasing gratitude. Using an experience sampling methodology, Olson et al. (2019) showed that prayer and meditation significantly contributed to an increase in gratitude during the three-week study period.

#### Gratitude in 12-Step Programs

Mutual-aid groups, specifically, 12-step programs, provide a medium for learning about gratitude. The program’s 10th step, which recommends a review of each day, suggests stopping to appreciate the daily “blessings received” (AA World Services, 1953, p. 95). A frequent recommendation in 12-step communities is to “make a gratitude list,” especially when feeling negative emotions.

Krentzman (2019) conducted a grounded theory study of the theme of gratitude in the writings of Alcoholics Anonymous’ co-founder, Bill Wilson, and found that, when addressing gratitude in his writings, Wilson described the AA program and recovery as gifts that come not from one’s own self, but from benefactors. The benefactors described in Wilson’s writings are God, or one’s higher power, and individuals who support a person’s recovery including other AA members, medical professionals, clergy, and family members. Gifts from these benefactors inspire the AA member to reciprocate, to give back in a spirit of gratitude for what they have received, by seeking and doing their higher power’s will and by carrying the message of recovery to others. In this way, in Wilson’s writings, spirituality and gratitude are closely aligned.

#### How Gratitude Supports Well-Being During Addiction Recovery

The theory of the maintenance of behavior change shows how gratitude can increase satisfaction with life in recovery and thereby support abstinence. This theory states that the maintenance of behavior change engages different psychological processes than the initiation of behavior change (Rothman et al., 2016). For the maintenance of behavior change, a person, whether they realize it or not, compares life now, after having made the change, to life before making the change. The person makes this comparison regularly and must repeatedly determine that “now” is better than “then” to continue the hard work of maintaining the change. A gratitude practice can amplify what is going right in recovery now, thereby increasing satisfaction with life in recovery, so that any comparison to active addiction results in the determination that now is better than then.

Gratitude can also enhance satisfaction with life in recovery in the ways that it enhances interpersonal relationships. Some scholars have stated that gratitude occurs when a person has benefited from the actions of another person or entity. Having received a gift, an individual is inspired to reciprocate (Emmons & Crumpler, 2000) and do something good for the benefactor or another person (Bartlett & DeSteno, 2006). This interpersonal reciprocity has led some scholars to conclude that gratitude is essential to everyday relationships (Algoe, 2012; Bartlett & DeSteno, 2006). Therefore, a gratitude practice can improve interpersonal relationships, which are essential to satisfaction with life in recovery.

Gratitude can improve mood through physiological and psychological pathways. Scientists have found that changes take place in the brain as the result of active addiction (Ahmed et al., 2002; Koob, 2008). These changes persist into early recovery and make it harder for the individual to feel positive emotions and easier for them to experience stress. Therefore, in early recovery, a gratitude practice could help train the brain to feel positive emotions in response to everyday pleasures in the absence of addictive behavior.

Two psychological principles describe the human propensity toward negativity, which can worsen mood during recovery from addiction. The negativity bias explains the ways in which negative thoughts, emotions, and experiences have a greater impact on us than positive or neutral experiences (Baumeister et al., 2001; Rozin & Royzman, 2001). This tendency is thought to have an evolutionary purpose, to aid survival; but in current times, it can result in a preponderance of negative focus, upsetting emotions, and fear. A gratitude practice can help a person in recovery give greater attention to what is going well in order to balance the negativity bias with positive thoughts and emotions.

The concept of the hedonic treadmill can also be applied to the role of gratitude in addiction recovery. The hedonic treadmill describes that each person has a set level of positive or negative mood (Diener et al., 2006). When good or bad things happen, in general, a person experiences a rise or fall in their average mood state. The person ultimately adapts to the new circumstance with a return to their baseline level of mood. This explains why improvements in short-term happiness are not lasting; individuals become accustomed to them. Gratitude can play a role here for the person in recovery – it can reawaken the original good feelings an individual experienced when a good circumstance was new, fostering a positive mood.

Gratitude’s ability to improve mood via physiological and psychological pathways supports recovery from addiction in the ways that it counters negative emotions. Gratitude has been shown to be associated with less stress (LaBelle & Edelstein, 2018) and less psychological distress (Krieger et al., 2023) among members of 12-step programs. Further, negative emotion in recovery has been found to be a significant trigger for relapse (Sliedrecht et al., 2019) including individuals with CSBD (Wordecha et al., 2018).

Some authors have emphasized emotional dysregulation in individuals with CSBD (Lew-Starowicz et al., 2020). A study by Wordecha et al. (2018) suggests that binge pornography use and masturbation are ways to regulate mood and stress and can be treated as a short-term mechanism of coping with negative emotions, which in turn lead to cumulative negative emotions, such as shame, loneliness, disgust, guilt, anger, sadness, or anxiety. Developing gratitude during SA meetings may help SA members to self-regulate emotionally without resorting to sexual acting out.

### Hypotheses

In the current study, we examine the direct and indirect relationships between the number of 12 steps completed, spiritual growth, gratitude, and subjective well-being among SA members. Based on the theoretical and empirical background, we expect that:

#### Hypothesis 1

**(H1)**: In a sample of SA members, the number of 12 steps completed is positively related to life satisfaction and positive affect and negatively related to negative affect.

#### Hypothesis 2

**(H2)**: In a sample of SA members, the number of 12 steps completed is positively related to spiritual growth.

#### Hypothesis 3

**(H3)**: In a sample of SA members, the number of 12 steps completed is directly and positively related to gratitude, and this effect is partially mediated by spiritual growth.

#### Hypothesis 4

**(H4)**: In a sample of SA members, gratitude is positively related to life satisfaction and positive affect and negatively related to negative affect.

Furthermore, taking all of the above direct and indirect relationships together, we formulate the following hypothesis concerning the 12-step pathway of recovery:

#### Hypothesis 5

**(H5)**: In a sample of SA members, the number of 12 steps completed is positively related to spiritual growth, which indirectly, through gratitude, predicts greater satisfaction with life, higher levels of positive affect, and lower levels of negative affect.

## Materials and Methods

### Participants

Table 1 presents the sociodemographic characteristics of the study sample. The sample consisted of 80 individuals attending SA meetings in Poland, including 72 men and 8 women. The average age of study participants was 38.96 years (*SD* = 10.56; min. = 22, max. = 68). Most participants graduated from universities (78.8%). About half of the sample (51.3%) were single, and 45% of the participants were married. Nearly half of the participants (46.3%) had at least one child. Most participants declared to be Roman Catholic (82.5%).

**Table 1:** Sociodemographic characteristics of the sample

Gender	Men	72 (90%)
	Women	8 (10%)
Age (years; M ± SD)	38.96 (10.56)	
Educational level	Vocational	2 (2.4%)
	Secondary	15 (18.8%)
	Higher	63 (78.8%)
Marital status	Single	41 (51.3%)
	Married	36 (45.0%)
	Separated	2 (2.5%)
	Divorced	1 (1.2%)
Child	No	43 (53.7%)
	Yes	37 (46.3%)
Religious denomination	Roman Catholicism	66 (82.5%)
	Without denomination	9 (11.2%)
	Slavic religion	2 (2.5%)
	Jehovah’s Witnesses	2 (2.5%)
	Agnostic	1 (1.3%)
Previous use of psychological or therapeutic services	No	6 (7.0%)
	Yes	74 (93.0%)
Current use of psychological or therapeutic services	No	44 (55.0%)
	Yes	36 (45.0%)
Number of months in Sexaholics Anonymous (M ± SD)	47.48 (32.86)	
Duration of sexual abstinence (months; M ± SD)	20.95 (28.64)	

*Note*. *M* = mean; *SD* = standard deviation

The vast majority of the sample (93.0%) had used psychological or psychotherapeutic services at least once in their lives, whereas nearly half of the participants (45.0%) were currently using these services. On average, the participants had been attending SA meetings for nearly four years (*M* = 47.48 months, *SD* = 32.86).

### Measures

#### Number of 12 Steps Completed

The number of 12 steps completed was measured using the item from the Alcoholics Anonymous Involvement Scale (AAIS; Tonigan et al., 1996). The respondents were asked about the number of steps they had already completed in the 12-step SA program (from 0 to 12). The 12 steps of SA are listed in Table 2. The number of 12 steps completed—treated as a measure of the advancement in mutual-aid groups for addictions—was used in many addiction studies, which identified it as one of the indicators of involvement in these groups (Beasley et al., 2023; Tonigan et al., 1996).

**Table 2:** The 12 steps of Sexaholics Anonymous

Number of the step	Content
1	We admitted that we were powerless over lust—that our lives had become unmanageable.
2	Came to believe that a Power greater than ourselves could restore us to sanity.
3	Made a decision to turn our will and our lives over to the care of God as we understood Him.
4	Made a searching and fearless moral inventory of ourselves.
5	Admitted to God, to ourselves, and to another human being the exact nature of our wrongs.
6	Were entirely ready to have God remove all these defects of character.
7	Humbly asked Him to remove our shortcomings.
8	Made a list of all persons we had harmed, and became willing to make amends to them all.
9	Made direct amends to such people wherever possible, except when to do so would injure them or others.
10	Continued to take personal inventory and when we were wrong promptly admitted it.
11	Sought through prayer and meditation to improve our conscious contact with God as we understood Him, praying only for knowledge of His will for us and the power to carry that out.
12	Having had a spiritual awakening as the result of these Steps, we tried to carry this message to sexaholics, and to practice these principles in all our affairs.

*Note*. Adapted from *Sexaholics Anonymous (SA)*, by Sexaholics Anonymous, 1989. Copyright 1989–2002 by SA Literature

#### Spiritual Growth

Spiritual growth was measured with the short version of the Daily Spiritual Experiences Scale (DSES; Underwood and Teresi, 2002), which consists of 6 out of the original 16 items of this tool (example item: “I am spiritually touched by the beauty of creation.”). The short version of the DSES has been successfully used in several studies with Polish samples (Charzyńska et al., 2021; Wnuk, 2023). Charzyńska et al. (2021) found the unidimensionality of the short version of the DSES in a Polish sample of young adults. In the current study, 6 items of the DSES explained 60.1% of the variance in spiritual growth. Each item of the DSES is assessed with a six-point Likert scale (1 = “many times a day,” 2 = “every day,” 3 = “most days,” 4 = “some days,” 5 = “once in a while,” and 6 = “never or almost never”). The total level of daily spiritual experiences is calculated by summing up the responses, which need to be reverse-scored before. Higher scores reflect a higher frequency of daily spiritual experiences (see Underwood, 2011), which can be interpreted as a marker of spiritual growth.

#### Gratitude

Trait gratitude was measured with the Polish adaptation (Kossakowska & Kwiatek, 2014) of the Gratitude Questionnaire (GQ-6; McCullough et al., 2002). The GQ-6 consists of 6 items (e.g., “I am grateful to a wide variety of people.”), rated on a 7-point Likert scale (from 1 = “strongly disagree” to 7 = “strongly agree”). The scale is homogenous, measuring a single factor called “gratitude.” Two items are reverse-scored before calculating the total score. The level of gratitude is obtained by summing up all the items.

#### Well-Being

We assessed the overall concept of well-being by its two primary component parts: satisfaction with life and affect as follows.

##### Satisfaction with Life

The Polish version (Jankowski, 2015) of the Satisfaction with Life Scale (SWLS; Diener et al., 1985) was used to measure life satisfaction, defined as a cognitive component of well-being. This measure has a unidimensional structure and consists of five items (e.g., “In most ways my life is close to my ideal.”), which are assessed using a 7-point Likert scale (from 1 = “strongly disagree” to 7 = “strongly agree”). Higher scores indicate higher levels of life satisfaction.

##### Positive and Negative Affect

Affect was measured with the Positive and Negative Affect Schedule (PANAS; Crawford and Henry, 2004). In the current study, the short version of the PANAS was used by selecting the five strongest loading items for positive affect (e.g., enthusiasm) and negative affect (e.g., fear) out of the 20-item version of this tool (Wnuk & Marcinkowski, 2014). In the current study, the two-factor structure of the PANAS was supported by the results of the principal component analysis, which extracted two components, explaining 34.33% (negative affect) and 31.49% (positive affect) of the variance of the affectivity construct. The PANAS items are scored on a 5-point Likert scale (from 1 = “a little or none” to 5 = “very frequently”). Higher scores indicate experiencing positive/negative affect more frequently.

### Procedure

The study was conducted between February 2020 and January 2021 in Poland. All procedures were carried out in accordance with the Declaration of Helsinki. The study protocol was accepted by the Ethics Committee at the University of Silesia in Katowice (KEUS 123/05.2021). To collect a sample, the research team member sent an email to several members of SA in Poland that they were familiar with, requesting them to disseminate the information about the study along with the survey link to other SA members. The inclusion criteria involved being at least 18 years old and attending SA meetings in Poland. The online survey was preceded by information about the research purpose, anonymous and voluntary participation in the study, the approximate duration of the survey, and the right to withdraw from the study at any time without any consequences. All participants gave their online consent before completing the survey. No incentives were offered to participants.

### Statistical Analysis

Before testing the sequential mediation model, we assessed common method bias using Harman’s one-factor test. The total variance extracted by one factor exceeding 50% indicates a serious common method deviation. In addition, we calculated the variance inflation factor (VIF) for the predictors to examine whether there were not too high correlations between predictors. Values of VIF higher than 5 suggest that multicollinearity occurs in data (James et al., 2017).

Next, we tested the assumption of multivariate normality by calculating Mardia’s (1970) coefficient of multivariate kurtosis and its critical ratio. The value of a critical ratio lower than 5 suggests that the data can be deemed normally distributed (Bentler, 2005). In the last step of preliminary analysis, we calculated descriptive statistics, reliability coefficients, and zero-order correlations between the study variables.

The mediation model was tested using a path analysis based on the maximum likelihood estimation of structural equation modeling (SEM). Model fit was assessed using several fit indices: the model χ^2^, the minimum discrepancy*/*degrees of freedom (χ^2^/df), the comparative fit index (CFI), goodness-of-fit index (GFI), Tucker-Lewis index (TLI), normed fit index (NFI), incremental fit index (IFI), the root mean square error of approximation (RMSEA), and standardized RMR (SRMR). *P*-value for χ^2^ > 0.05, χ^2^/df < 3, CFI, GFI, TLI, NFI, and IFI ≥ 0.95, and RMSEA and SRMR < 0.08 suggest a good model fit (Hair et al., 2010; Kline, 2005). In addition, we used the Bollen-Stine bootstrapping procedure to calculate a bootstrap-adjusted *p*-value of an empirical SEM model (Bollen & Stine, 1992).

To test the significance of the indirect effects in the mediation model, we used the bootstrapping method, which is widely acknowledged as the best available option to test the indirect effects (Preacher & Hayes, 2004). We used 95% confidence intervals (CI) based on 5,000 bootstrap replications. If a coefficient does not include 0 in its 95% confidence interval, it can be deemed statistically significant. The effect size for indirect relationships was calculated as a completely standardized indirect effect (Preacher & Kelley, 2011). The model was controlled for age, considering previous findings supporting the predictive role of this variable in subjective well-being (Baird et al., 2010), gratitude (Chopik et al., 2019), and spirituality (Zimmer et al., 2016). We used the criterion of *p* < .2 for the inclusion of covariates, as suggested by Maldonado and Greenland (1993). All calculations were performed using AMOS version 27.0 (Arbuckle, 2020) and IBM SPSS version 27.0 (IBM Corp., 2020).

## Results

### Preliminary Analysis

The value of Harman’s single factor test (29.45%) suggested that the problem of common method bias did not occur in our data. There were no predictors with the values of VIF exceeding 5 (maximum VIF, noted for spiritual growth, was 1.56); thus, the multicollinearity problem was not likely to exist for our data. The value of the critical ratio was − 0.46, which indicates that the assumption of multivariate normality was met.

Table 3 presents the descriptive statistics, reliability of the measures, and correlations between the variables. On average, SA members completed 7 of the 12 steps (*M* = 6.99, *SD* = 4.17). The number of 12 steps completed correlated positively with spiritual growth (*r* = .24; *p* = .036), life satisfaction (*r* = .28; *p* = .012), and inversely with negative affect (*r* = − .38; *p* < .001). Spiritual growth was positively related to gratitude (*r* = .54; *p* < .001), life satisfaction (*r* = .45; *p* < .001), and positive affect (*r* = .35; *p* = .002), and inversely to negative affect (*r* = − .32; *p* = .003). Life satisfaction was positively related to positive affect (*r* = .56; *p* < .001). Reliability measured with McDonald’s omega was satisfactory for all measurement tools (see Table 3).

**Table 3:** Descriptive statistics, reliability coefficients, and zero-order correlations between the study variables

Variables	(1)	(2)	(3)	(4)	(5)	(6)	(7)
(1) Number of 12 steps completed	1						
(2) Spiritual growth	.24*	1					
(3) Gratitude	.18	.54***	1				
(4) Life satisfaction	.28*	.45***	.44***	1			
(5) Positive affect	.19	.35***	.45***	.56***	1		
(6) Negative affect	−.38***	−.32**	−.15	−.18	.04	1	
(7) Age	.07	−.02	−.13	−.15	−.20	−.04	1
M	6.99	23.99	34.11	20.59	15.88	16.15	38.96
SD	4.17	5.91	5.75	5.30	4.23	4.48	10.56
Scale range	0–12	6–36	7–42	5–35	5–25	5–25	22–68
McDonald’s omega	–	.87	.85	.82	.86	.87	–

*Note. M* = mean, *SD* = standard deviation; *p < .05, **p < .01, ***p < .001

### Path Analysis

The values of model fit criteria indicated that the model has a good fit to data: χ^2^(5) = 5.27; *p* = .38; CMIN/df = 1.05; CFI = 0.997; GFI = 0.982; TLI = 0.989; NFI = 0.957; IFI = 0.998, RMSEA = 0.026 (90% CI [0.000, 0.160]), and SRMR = 0.038. Although the upper CI for the RMSEA exceeded the recommended value of 0.08, this is a common issue in models with low degrees of freedom and small sample size (Kenny et al., 2015). The good model fit was further supported by the insignificant bootstrap-adjusted *p*-value (0.43).

**Fig. 1:**
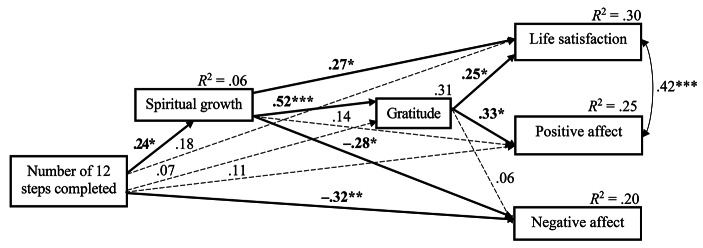
Results for path analysis: Sequential mediation model with standardized coefficients. *Note*. Bold continuous lines indicate the significant relationships, whereas dotted lines indicate the insignificant ones. The model was adjusted for age. *R*^2^ = explained variance. *p < .05, **p < .01, ***p < .001

The standardized coefficients for the path model are presented in Fig. 1. As for direct effects, the number of 12 steps completed was positively related to spiritual growth (β = 0.24; 95% CI [0.010, 0.466]; *p* = .036) and inversely to negative affect (β = −0.32; 95% CI [− 0.528, − 0.097]; *p* = .004), but not to gratitude (β = 0.07; 95% CI [− 0.153, 0.253]; *p* = .561), life satisfaction (β = 0.18; 95% CI [− 0.007, 0.372]; *p* = .061), or positive affect (β = 0.11; 95% CI [− 0.086, 0.301]; *p* = .273). Spiritual growth correlated positively with gratitude (β = 0.52; 95% CI [0.319, 0.723]; *p* < .001) and life satisfaction (β = 0.27; 95% CI [0.047, 0.485]; *p* = .015) and inversely with negative affect (β = −0.28; 95% CI [− 0.510, − 0.058]; *p* = .014). The relationship between spiritual growth and positive affect (β = 0.14; 95% CI [− 0.131, 0.363]; *p* = .301) was insignificant. Gratitude was positively related to life satisfaction (β = 0.25; 95% CI [0.047, 0.416]; *p* = .016) and positive affect (β = 0.33; 95% CI [0.058, 0.568]; *p* = .024) but not to negative affect (β = 0.06; 95% CI [− 0.192, 0.330]; *p* = .661).

All indirect effects with 95% confidence intervals are shown in Table 4. The number of 12 steps completed was indirectly related to gratitude through spiritual growth (β = 0.12; 95% CI [0.016, 0.287]; *p* = .023). Moreover, the significant indirect effects through spiritual growth were noted for the relationships between the number of 12 steps completed and life satisfaction (β = 0.06; 95% CI [0.007, 0.176]; *p* = .023) and between the number of 12 steps completed and negative affect (β = −0.07; 95% CI [− 0.202, − 0.008]; *p* = .020).

**Table 4:** Indirect effects with 95% confidence intervals

	Estimate	95% lower bound	95% upper bound	p-value
**Number of 12 steps completed→spiritual growth→gratitude**	**0.12**	**0.016**	**0.287**	**0.023**
**Number of 12 steps completed→spiritual growth→life satisfaction**	**0.06**	**0.007**	**0.176**	**0.023**
Number of 12 steps completed→spiritual growth→positive affect	0.03	−0.020	0.138	0.192
**Number of 12 steps completed→spiritual growth→negative affect**	**−0.07**	**−0.202**	**−0.008**	**0.020**
Number of 12 steps completed→gratitude→life satisfaction	0.02	−0.030	0.081	0.426
Number of 12 steps completed→gratitude→positive affect	0.02	−0.039	0.109	0.345
Number of 12 steps completed→gratitude→negative affect	0.00	−0.016	0.072	0.542
**Spiritual growth→gratitude→life satisfaction**	**0.13**	**0.029**	**0.260**	**0.011**
**Spiritual growth→gratitude→positive affect**	**0.17**	**0.033**	**0.369**	**0.018**
Spiritual growth→gratitude→negative affect	0.03	−0.100	0.178	0.637
**Number of 12 steps completed→spiritual growth→gratitude→life satisfaction**	**0.03**	**0.006**	**0.096**	**0.014**
**Number of 12 steps completed→spiritual growth→gratitude→positive affect**	**0.04**	**0.006**	**0.131**	**0.018**
Number of 12 steps completed→spiritual growth→gratitude→negative affect	0.01	−0.022	0.062	0.478

*Note*. All coefficients were standardized. The effect is deemed significant when the confidence interval does not contain 0. All significant indirect effects were bolded. The model was controlled for age

In the last step, we tested the chains of relationships between the number of 12 steps completed as an antecedent of spiritual growth, gratitude, and well-being indicators. The indirect effects of the number of 12 steps completed through the sequential path of spiritual growth—gratitude were significant for life satisfaction (β = 0.03, 95% CI [0.006, 0.096]; *p* = .014), and positive affect (β = 0.04, 95% CI [0.006, 0.131]; *p* = .018). According to the interpretation of completely standardized indirect effects suggested by Preacher and Kelley (2011), all significant specific indirect effects noted in this study can be considered low to medium. The proportion of variance explained (*R*^2^) was 0.06 for spiritual growth, 0.31 for gratitude, 0.30 for life satisfaction, 0.25 for positive affect, and 0.20 for negative affect.

## Discussion

### The 12-Step Pathway to Well-Being

The purpose of this study was to examine the mechanisms underlying the relationship between the number of 12 steps completed and well-being and to test the role of spiritual growth and gratitude in these relationships. Partially consistent with H1, the number of 12 steps completed was inversely related to negative affect but was not related to either life satisfaction or to positive affect. This result may suggest that the 12-step program directly helps the SA members decrease the intensity of negative feelings. The explanation of this result may lie in the content of the 12-step program for addictions, in which the need to let go of resentment, bitterness and grudges for recovery is emphasized (AA, 2001; SA, 1989). In accordance with H2, the number of steps completed was positively related to spiritual growth, suggesting that with the completion of steps, SA members start to experience spirituality in everyday situations.

Although the number of 12 steps completed was not directly related to gratitude, it was indirectly related to this moral virtue through spiritual growth (H3 partially supported). This result supports the findings of previous studies, which noted the beneficial role of spirituality in increasing gratitude (Hardy et al., 2014; Krause & Hayward, 2015; Olson et al., 2019; Rosmarin et al., 2016; Tsang et al., 2012; Lambert et al., 2009).

Gratitude correlated positively with positive affect and life satisfaction but was not related to negative affect (H4 partially supported). This result is consistent with previous research, in which gratitude has been noted to be positively related to positive affect (Sun & Kong, 2013; Wood et al., 2008, 2010). Our finding corroborates with the broaden-and-build theory of positive emotions, which views gratitude as broadening thought repertoires and building a variety of personal and social resources (Fredrickson & Joiner, 2018). Gratitude may also promote life satisfaction by deepening relationships, providing social support, improving optimism and self-efficacy, and helping individuals to cope with adversity (Grant & Gino, 2010; Puente-Díaz & Cavazos-Arroyo, 2022; Robustelli & Whisman, 2018; Wood et al., 2008, 2010). Our findings show that gratitude provides benefits to SA members—benefits that support their recovery.

The lack of a significant relationship between gratitude and negative affect is not surprising given the fact that previous research on this topic is inconclusive; some studies noted an inverse relationship between gratitude and negative affect (Froh et al., 2011; McCullough et al., 2002; Wood et al., 2008), whereas others demonstrated that these two variables are not significantly related to each other (Datu et al., 2022; Froh et al., 2009; Grant & Gino, 2010; Puente-Díaz & Cavazos-Arroyo, 2019). In the context of SA, many individuals with CSBD may suffer from serious trauma and have a history of parental neglect or abuse (Slavin et al., 2020), which may be connected with high levels of grudges and revenge in this population. The relationship between gratitude and negative affect in individuals with CSBD may be thus moderated by the levels of forgiveness of others. Specifically, gratitude can be related to lower negative affect, but only if the person forgives their perpetrators. Otherwise, the disposition to gratitude may be insufficient to alleviate the negative emotions that SA members combat. Some individuals with CSBD may involve in upward counterfactual thinking (e.g., How much my life could have been better if I had not been harmed by others?), which can increase negative emotions connected with holding grudges against perpetrators, in this way blocking the potential of gratitude for reducing negative emotions (see Bernabe-Valero et al., 2021). This topic needs further investigation, as it may be a fruitful avenue of exploration of the interactions between gratitude and forgiveness.

The results of testing H5 showed the significant effects of the number of 12 steps completed on life satisfaction and positive affect (but not on negative affect) through the paths of spiritual growth and gratitude, respectively (H5 partially supported). Involvement in a 12-step program can be considered a meaning-making system (Wnuk, 2021) consisting of a cognitive-emotional-behavioral framework that determines one’s perception, interpretation, and behaviors, and creates a ground for spiritual growth. As a result of this meaning-making system, the appreciation of the gift of recovery, which is perceived as coming from a higher power and other members of mutual-aid groups, appears (Krentzman, 2019). Gratitude, in turn, encourages each SA member to carry the message of recovery to other SA members and to serve as a sponsor to repay their higher power and other SA members for all intangible goods and support they receive during the recovery process. Involvement in this process of feeling grateful and then being inspired by gratitude to serve others may foster a feeling of usefulness and good self-esteem; it may strengthen interpersonal bonds and help people in SA stay abstinent and have a happy and good life in recovery.

The results of the study also suggest that for improving life satisfaction and increasing and sustaining positive emotions, nurturing the moral virtue of gratitude of individuals with CSBD is recommended. Indeed, in the current study, gratitude directly predicted life satisfaction, positive affect but not negative affect. By contrast, the number of 12 steps completed was directly and negatively related to negative affect, but it was only indirectly (i.e., through spiritual growth and gratitude) related to positive well-being indicators (i.e., life satisfaction and positive affect). These findings corroborate with those of positive psychology when it postulates that the lack of pathology is not the same as well-being (Jans-Beken et al., 2018; Keyes, 2005): the mechanisms leading to reducing harmful behaviors like CSB, or diminishing negative affect that may trigger CSB, differ from the mechanisms underlying positive and desirable states.

From the practical point of view, since benefits were found for practicing the 12 steps, individuals with CSBD using other forms of treatment, such as pharmacotherapy or psychotherapy, should be encouraged to attend SA meetings as a supplementary form of support, which can help them live a more satisfying and happy life. Therapists should consider incorporating parts of the 12-step program, such as spirituality and gratitude, into the psychotherapeutic programs and interventions to support sexual abstinence and prevent relapse. Moreover, more information about SA as an effective support method for CSBD patients should be distributed among patients, regardless of their religious beliefs.

### Limitations

The study has some limitations that should be discussed. First, due to the cross-sectional character of the data, no unambiguous conclusions about the directionality of the relationships can be drawn from the study. Longitudinal studies monitoring the cascading effects of involvement in SA and other mutual-aid groups for CSBD on spiritual growth, gratitude, and their relationships with subjective well-being are highly recommended. Nevertheless, the model was based on well-grounded empirical and theoretical premises, mostly from the fields of psychology of religion and spirituality and positive psychology.

Second, due to the snowball method, we could not calculate the response rate, which limits the generalizability of the results. However, reaching out to SA members in other ways would have been difficult, especially due to the large dispersion of SA groups across the country and the restrictions related to the COVID-19 pandemic. Moreover, most of our respondents were men with a higher education level. Studies in other mutual-aid groups for CSBD with more sociodemographically diversified samples are needed.

Third, the exogenous variable in our model (i.e., the number of 12 steps completed) was measured only with a single item, which may have limited the variability of responses to some degree. The number of 12 steps completed as an indicator of the advancement in the SA program has been used in previous research, e.g., in a study by Efrati and Gola (2018), and we considered it adequate for the purpose of the current study. Nevertheless, future studies may benefit from using more complex measures of SA involvement.

Moreover, the content of one of the six items in the short version of the DSES (“I feel deep inner peace or harmony”) suggests that this measure is confounded by mental health indicators, which may have influenced the relationship between spiritual growth and well-being to some degree. Therefore, future studies exploring this relationship should consider using more pure measures of spiritual growth or the full version of the DSES.

Finally, our study was conducted in a highly religious context since more than 85% of respondents declared themselves to be religious, mostly Roman Catholic. Given that one of the roots of spiritual growth is religiousness (Puchalski & Guenther, 2012), a high number of religious individuals could have affected the results to some degree because being religious in a highly religious country is more beneficial for the individual than being religious in a more secular country (Stavrova et al., 2013). This shortcoming can be addressed by conducting cross-cultural studies involving more secular cultures and countries in which other religious denominations than Roman Catholicism are more common.

## Conclusion

This is the first study to examine the 12-step pathway to spiritual growth and gratitude and its relationships with subjective indicators of recovery in individuals attending SA meetings. Our study showed the direct relationship between the number of 12 steps completed and negative affect and the indirect relationships between the number of 12 steps completed, greater levels of spiritual growth and gratitude, and better well-being indicators. These findings support the beneficial role of the 12-step pathway through spiritual growth and gratitude in recovery from CSBD.
